# Material compatibility of guanidine thiocyanate for decontamination of nickel-titanium root canal instruments after potential exposure to prions

**DOI:** 10.3205/dgkh000548

**Published:** 2025-05-12

**Authors:** Anne-Maria Boldt, Walter J. Schulz-Schaeffer, Hicham Benkhai, Axel Kramer

**Affiliations:** 1 Sanitätsversorgungszentrum, Storkow, Germany; 2 Institute of Neuropathology, Medical Faculty of the Saarland University, Homburg, Germany; 3 Institute of Hygiene and Environmental Medicine, University Medicine, Greifswald, Germany

**Keywords:** guanidine thiocyanate, endodontic files, bending stiffness, torsional strength

## Abstract

**Aim:**

Due to the effectiveness of guanidine thiocyanate (GdnSCN) for the decontamination of prion protein aggregates, which are the causative agent of transmissible spongiform encephalopathy, the influence on the bending stiffness and torsional strength of endodontic nickel-titanium files should be tested to provide a potential alternative to single-use if necessary.

**Method:**

For the investigation, nitrite-titanium-coated EasyShape^®^ files of sizes 25.06 and 35.04 were placed in 6 M GdnSCN solution 8 times for 15 min each for decontamination in line with the manufacturer’s recommendation, with intermediate drying in each case. To simulate the worst case, the soaking time was extended to 12 h once. Both the bending stiffness and the torsional and fracture behavior were determined in accordance with DIN EN ISO 3630-1:2008-04.

**Results:**

Compared to the untreated control (n=12), decontamination with GdnSCN has no effect on the torsional strength or flexural rigidity of the tested endodontic instruments of sizes 25.06 and 35.04 (n=18 each) when used properly. On the other hand, the exposure time of 12 h reduced the bending moment and torsion angle of instrument size 25.06, while the material properties of size 35.04 files are not affected.

**Discussion:**

Based on the results, the maximum 8-fold application of 6 M GdnSCN solution for 15 min for decontamination of endodontic nickel-titanium files can be considered. Although the sterilization process has no influence on the fracture behavior, it is important to clarify for clinical practice what influence the overall reprocessing process has on the performance of the instruments.

## Zusammenfassung

**Zielsetzung:**

Auf Grund der Wirksamkeit von Guanidinthiocyanat (GdnSCN) zur Dekontamination von Prionproteinaggregaten, Erreger der transmissiblen spongiformen Enzephalopathie, sollte der Einfluss auf die Biegesteifigkeit und Torsionsfestigkeit endotischer Nickel-Titan-Feilen geprüft werden, um ggf. eine Alternative zum single-use zu haben.

**Methode:**

Für die Untersuchung wurden Nitrit-Titan-beschichtete EasyShape^®^-Feilen der Größen 25.06 und 35.04 zur Dekontamination gemäß Herstellerempfehlung 8mal je 15 min in 6 M GdnSCN-Lösung mit jeweiliger Zwischentrocknung eingelegt. Zur Simulation des worst case wurde die Einlegezeit auf einmalig 12 h verlängert. Sowohl die Biegesteifigkeit als auch das Torsions- und Frakturverhalten wurden gemäß DIN EN ISO 3630-1:2008-04 bestimmt.

**Ergebnisse:**

Im Vergleich zur unbehandelten Kontrolle (n=12) hat die Dekontamination mit GdnSCN bei sachgemäßer Anwendung keinen Einfluss auf die Torsionsfestigkeit und Biegesteifigkeit der geprüften Wurzelkanalinstrumente der Größen 25.06 und 35.04 (je n=18). Bei Überschreiten der Einwirkungszeit auf 12 h verringern sich dagegen bei der Instrumentengröße 25.06 das Biegemoment und der Verdrehwinkel, während Feilen der Größe 35.04 nicht in den Materialeigenschaften beeinflusst werden.

**Schlussfolgerung:**

Grundsätzlich kommt auf Grund der Ergebnisse die maximal 8fache Anwendung von 6 M GdnSCN-Lösung für 15 min zur Dekontamination von endodontischen Nickel-Titan-Feilen in Betracht. Obwohl der Sterilisationsprozess keinen Einfluss auf das Frakturverhalten hat, gilt es für die klinische Praxis zu klären, welchen Einfluss der Gesamtprozess der Aufbereitung auf die Leistung der Instrumente hat.

## Introduction

Root canal instruments are classified as relevant for the transmission of prions, the causative agents of transmissible spongiform encephalopathies (TSEs) – in humans Creutzfeldt-Jacob disease (CJD) and its variant vCJD [1], [2] – mainly due to direct contact with the peripheral branches of the trigeminal nerve. Iatrogenic transmission was first observed in 1974 [3] and can be caused by the use of neurosurgical instruments contaminated with prions, including root canal instruments [4], [5]. The peculiarity of TSE pathogens is their resistance to conventional disinfection and sterilization measures [6], [7]. This is due to the nature of the pathogens, namely misfolded, self-replicating proteins assembled into insoluble aggregates. Even at 600°C dry hot air exposure, residual infectivity remains at very high initial titers [8]. Therefore, since April 2007, the UK Department of Health has recommended that root canal instruments should only be used once [9]. This guideline was reaffirmed in 2013 [10]. As the exclusive use of single-use files is controversial, a questionnaire survey was conducted in 27 dental practices in Pretoria, South Africa in 2019. As a result, single-use endodontic files were used in 33.3%, mainly for economic reasons [11].

The aim of reprocessing medical devices is to prevent the transmission of pathogens, including TSE prions. With regard to the latter, reprocessing can be performed dependent on a recognizable (or suspected) risk of CJD/vCJD, e.g., diagnosis of possible or clinically probable CJD/vCJD or rapidly progressing dementia, or an unrecognizable disease risk. In order to minimize risk, it is essential to identify at-risk individuals and risk interventions based on the pathogen load of the affected tissue. Consequently, a medical assessment must be made before every elective procedure to determine whether there is a recognizable risk of a TSE, if necessary, with the help of a specialist. If the risk cannot be ruled out, either the product must be discarded or single-use files used, which contradicts the principle of sustainable handling, or a prion-inactivating reprocessing method must be selected. Effective means to this end are 24-hour soaking in 1–2 M sodium hydroxide solution, in 2.5 to 5% sodium hypochlorite solution for 24 h or in guanidine thiocyanate (GdnSCN) (3 M for 24 h, 4 M for 1 h or 6 M for 15 min). Instruments containing aluminum should not be disinfected in sodium hydroxide solution due to the risk of corrosion. Here, reprocessing in 4 M GdnSCN solution for 2x30 min is recommended [12]. The applicability of the available procedures should be clarified with the manufacturer of the instruments [13], [14]. If there is no risk of TSE, the method of choice after manual alkaline pre-cleaning is reprocessing in a washer-disinfector at 93°C with subsequent steam sterilization at 134°C and a holding time of 3 min [12].

Since GdnSCN is preferable to sodium hydroxide solution or sodium hypochlorite in suspected cases due to its material compatibility, this study tested its material compatibility for nickel-titanium root canal instruments.

## Method

### Materials tested 

Batch-clean nickel-titanium-coated EasyShape^®^ files (Gebr. Brasseler GmbH & Co. KG, Lemgo, Germany) sizes 25.06 and 35.04 were used for the study.

### Decontamination

Pre-cleaning, reprocessing in washer-disinfector and final steam sterilization were omitted in order to assess the effect of GdnSCN alone on the endodontic files.

In practice, GdnSCN decontamination should precede reprocessing. Care must be taken to ensure that the GdnSCN solution is thoroughly rinsed off before further reprocessing takes place. GdnSCN must not be mixed with acids! The solution must be disposed of in its own containers as hazardous waste. The solution can be used several times over a longer period of time. GdnSCN should only be handled with protective goggles.

Assuming that the instruments had been in contact with tissues of high infectivity, the files were placed in 6 M GdnSCN solution (Merck KGaA, Darmstadt, Germany) for 15 min and 7 replicates were performed. After each run, the files were rinsed with sterile distilled water and placed on disposable cellulose for 5 min to dry. This corresponds to the maximum frequency of use permitted by the manufacturer [14]. In addition, the root canal files were stored for 12 h in 6 M GdnSCN solution in a series of tests to determine the possible effects of exceeding the manufacturer’s instructions. 18 instruments were tested for each file size and 12 instruments for each control.

### Test equipment 

The bending behavior was tested using a device manufactured by ETH-Messtechnik GmbH (Gschwend, Germany) in accordance with DIN EN ISO 3630-1:2008-04 [15]. The measurement data was recorded and analyzed using software from IPS GmbH (Braunschweig, Germany) (version 1.0-10/94).

The device and transducer (Motor Cap Mecmesin GmbH, Schwenningen, Germany) used to test the torsional and fracture behavior in accordance with DIN EN ISO 3630-1:2008-04 [15] was type MC5G with a maximum rotation of 8.3 rpm. The device is equipped with sensor type MT-TS 50 Ncm “P”. The measured values were processed with CapGraph software (Mecmesin GmbH, Schwenningen, Germany; photos of the equipment in [16])

### Testing the bending behavior

The test was carried out in accordance with DIN EN ISO 3630-1:2008-04 [15]. Wire-cutting pliers were used to cut off the handle at the point where it is attached to the shaft of the root canal instrument. The test device was set so that only a maximum torsion angle of 45° was permitted. The instrument tip of the test specimen was clamped over a length of 3 mm in the jaws of the chuck perpendicular to the motor axis. The clamping jaws were then tightened. A driving pin is attached extraaxially to the rotating disk located on a drive shaft. The measuring device was moved on a ball-guided linear slide until the instrument to be measured was positioned above the drive pin. The motor was turned step by step in the direction until the driving pin touched the test specimen lightly and without tension. Before each test, the test device was calibrated to zero. During the test sequence, the motor turned the rotating disk with driving pin clockwise up to a maximum angle of rotation of 45°. The torque or bending moment applied during the test was recorded in Ncm and displayed in a line diagram.

### Testing the torsion and fracture behavior 

The test was carried out in accordance with DIN EN ISO 3630-1:2008-04 [15] as follows. Using wire-cutting pliers, the handle was cut off at the point where it is attached to the shaft of the root canal instrument. First, the transducer was calibrated to the torque range of the test specimen. Before the instrument to be measured was clamped in the chuck, which is located directly on the motor, it was checked for visible damage. The test specimen was then clamped in the chuck to a maximum length of 1 mm of the unmachined shaft and firmly fixed in the chuck. The measuring device was moved slowly on the ball-guided linear slide until the tip of the instrument on the test specimen protruded 3 mm into the brass clamping jaws. It was essential to ensure that the test specimen was straight and centered in the clamping jaws. Only when these requirements were met was the chuck tightened. Under certain circumstances, the clamping of the test specimen could exert a preload on the test specimen. This undesirable preload was gradually reduced to zero using the motor and the measuring indicator. The instruments were rotated clockwise. The measurement was completed when the instrument fractured. Using the above software, the measured maximum torsional moment [Ncm] and the torsion angle [°] were recorded and displayed in a line diagram. Both measuring devices, details of the test device and exemplary measurement curves are shown in [17].

### Statistical analysis 

The data sets were analyzed using Microsoft Excel (Microsoft Corporation, Redmont, USA) and SAS 9.3 software (SAS Institute Inc., Cary, NC, USA). The Kruskal-Wallis test was used to calculate significance. A value of p<0.05 was used as the significance threshold.

## Results

### Bending behavior 

**Figure 1:**
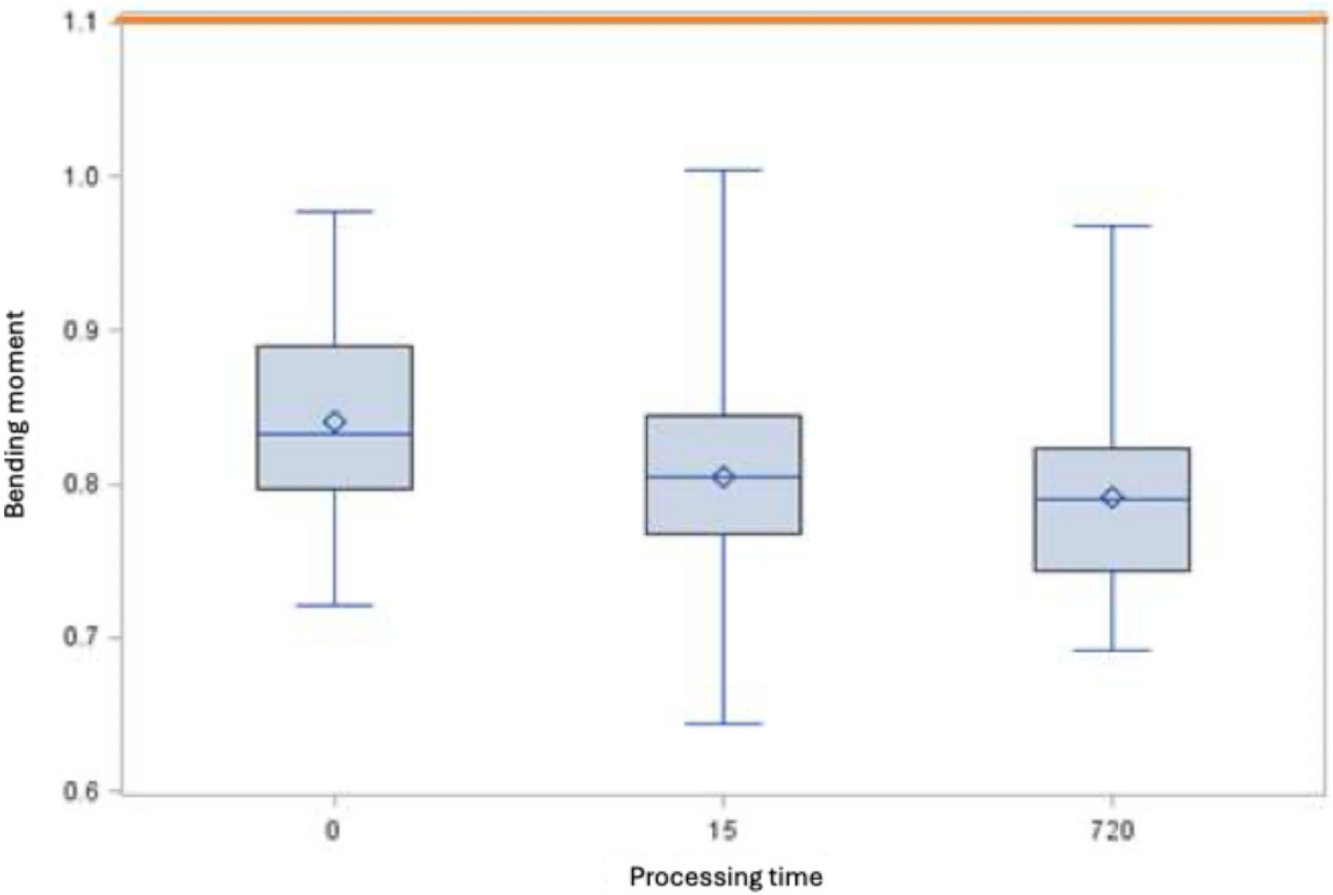
Bending moment [Ncm] of the root canal instruments size 25.06 (minimum, 25^th^ percentile, 50^th^ percentile, 75^th^ percentile, maximum and mean value ◊, the horizontal red line indicates the derived maximum value of 1.1 Ncm)

**Figure 2:**
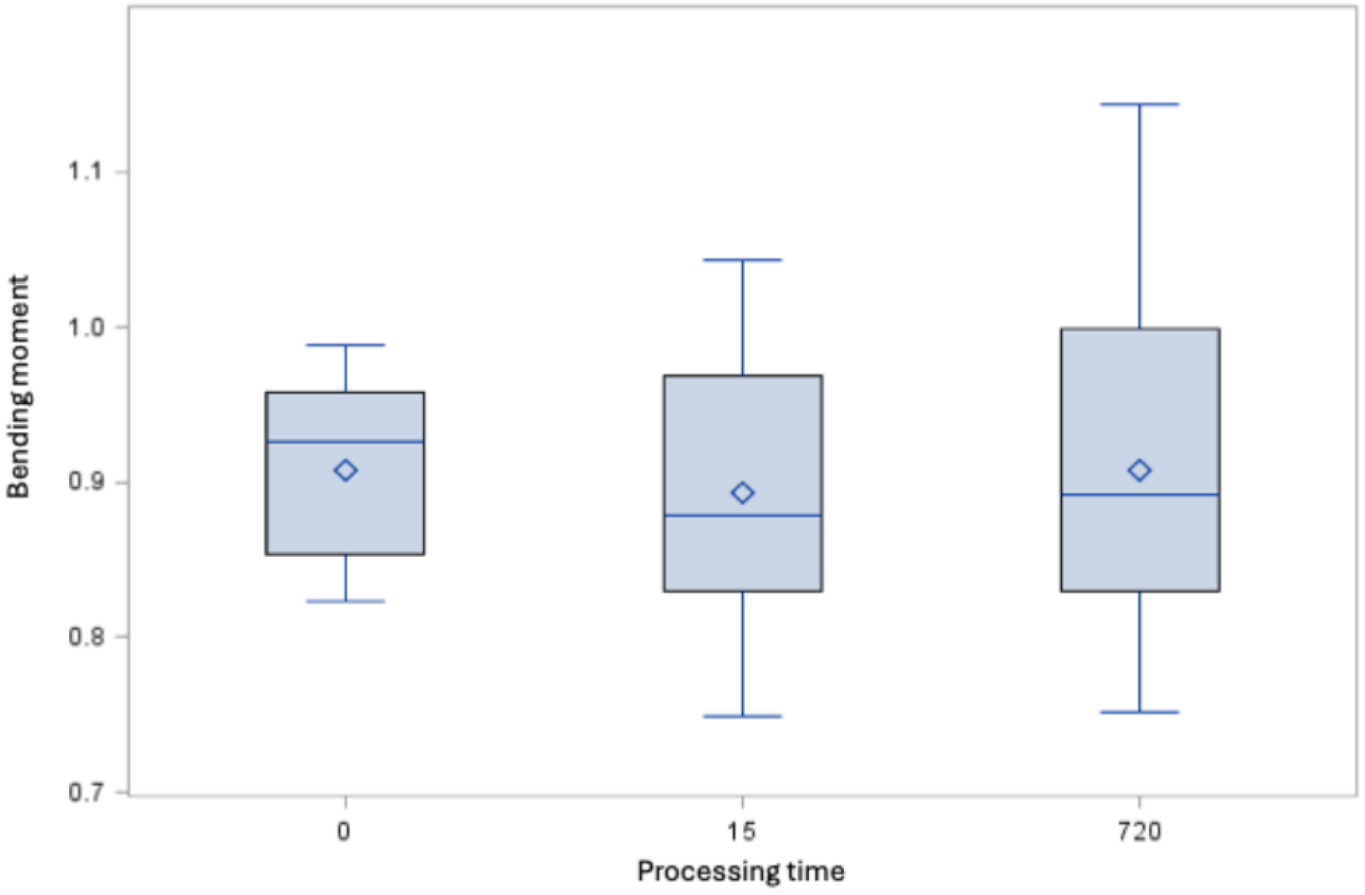
Bending moment [Ncm] of the root canal instruments size 35.04 (minimum, 25^th^ percentile, 50^th^ percentile, 75^th^ percentile, maximum and mean value ◊, the derived maximum value is 1.9 Ncm) after processing time of 15 and 720 min

DIN EN ISO [15] does not define any minimum requirements with regard to bending behavior for rotary nitrite-titanium root canal instruments with a taper >2%. However, if the results from the present study are compared with the minimum requirements according to the standard for instruments with a taper of 2%, all instruments treated with GdnSCN for 15 and 720 min as well as the untreated instruments of sizes 25.06 fulfill these requirements (Figure 1 (Fig. 1) and Figure 2 (Fig. 2)).

**Figure 3:**
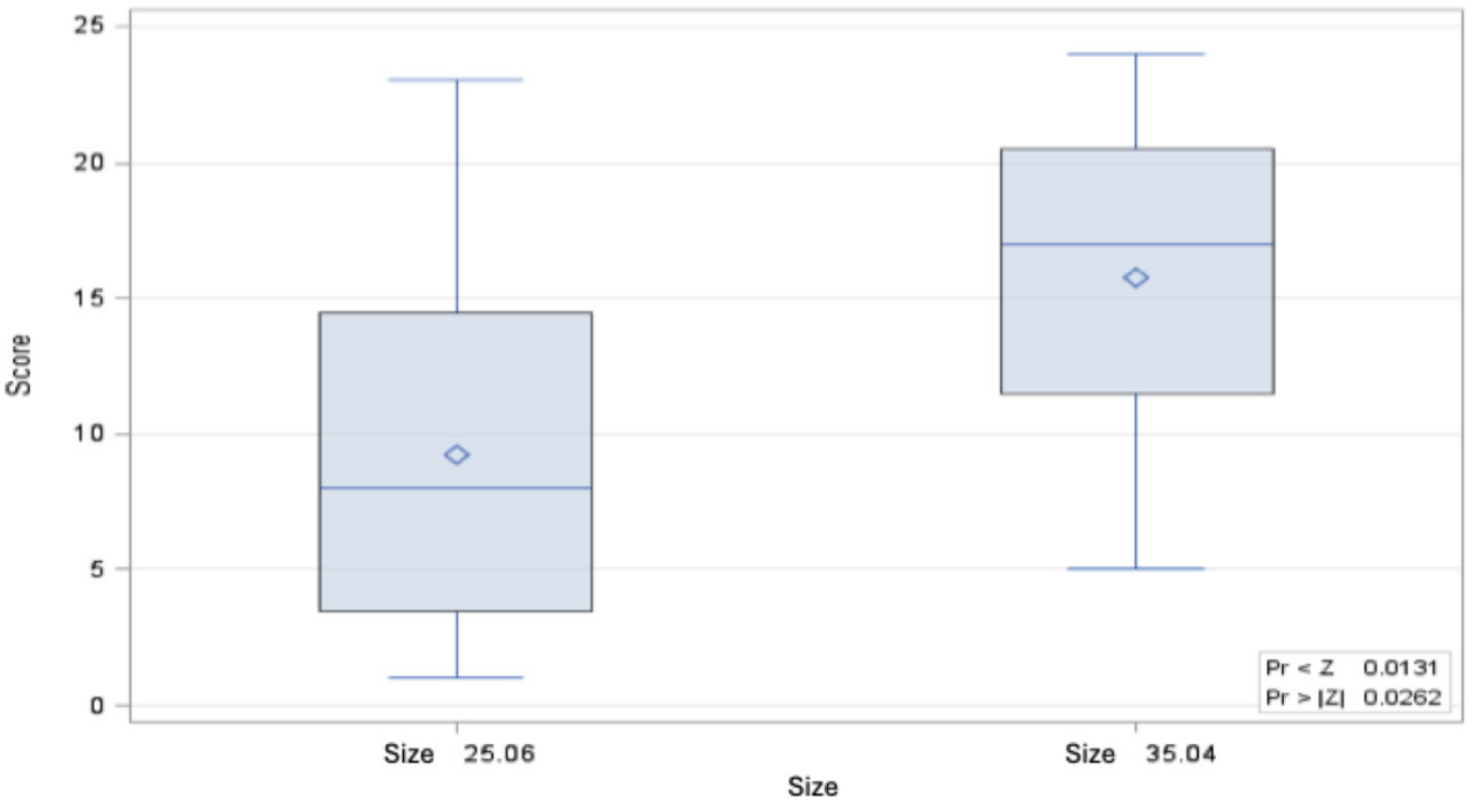
Distribution of Wilcoxon score values for the bending moment of controls of size 35.04 and 25.06 (p=0.0243) (minimum, 25^th^ percentile, 50^th^ percentile, 75^th^ percentile, maximum and mean value ◊)

**Figure 4:**
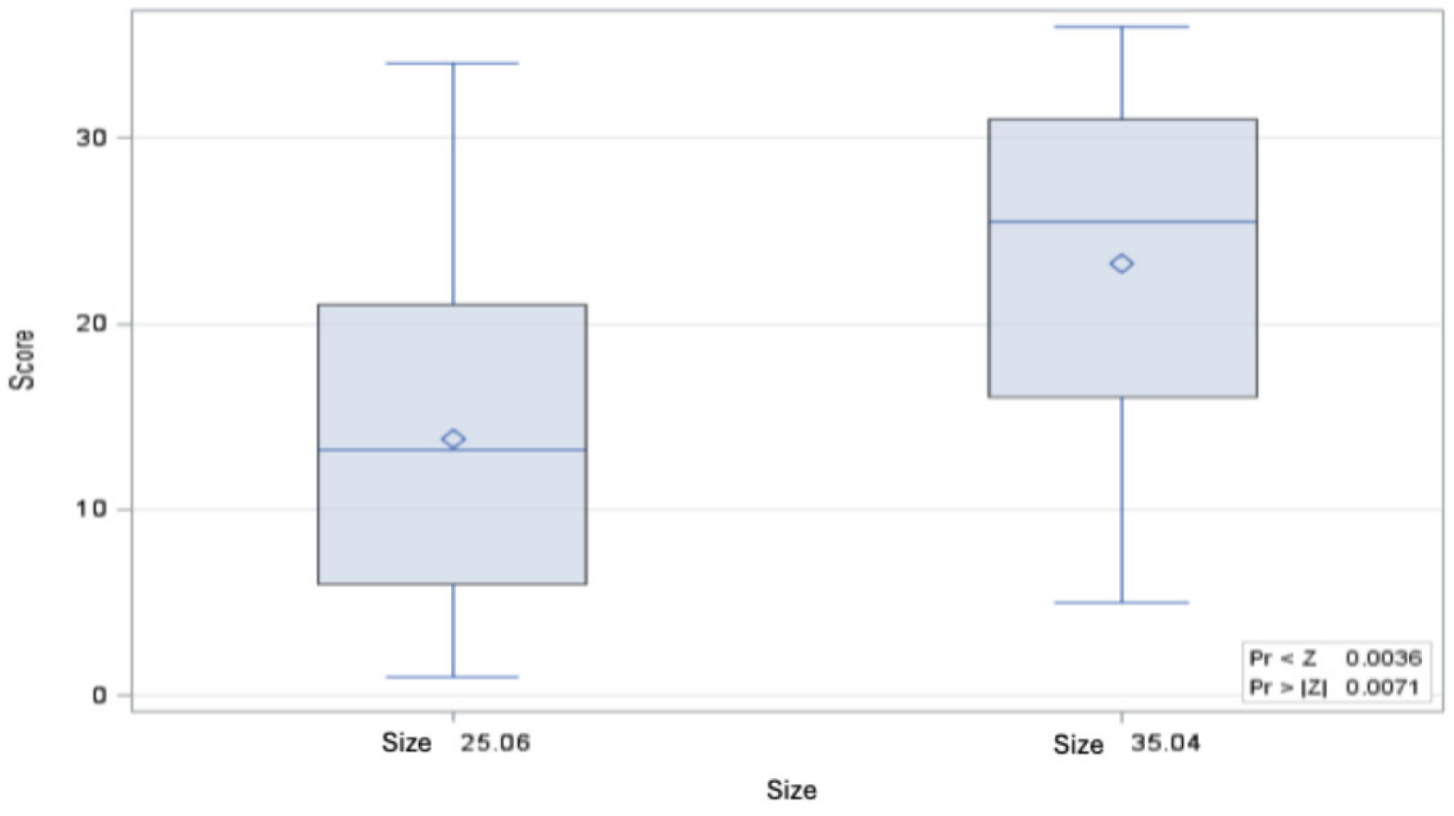
Distribution of Wilcoxon score values for the bending moment of size 35.04 untreated vs. treated 8 cycles of 15 min in 5 M GdnSCN solution (p=0.0490) (minimum, 25^th^ percentile, 50^th^ percentile, 75^th^ percentile, maximum and mean value ◊)

**Figure 5:**
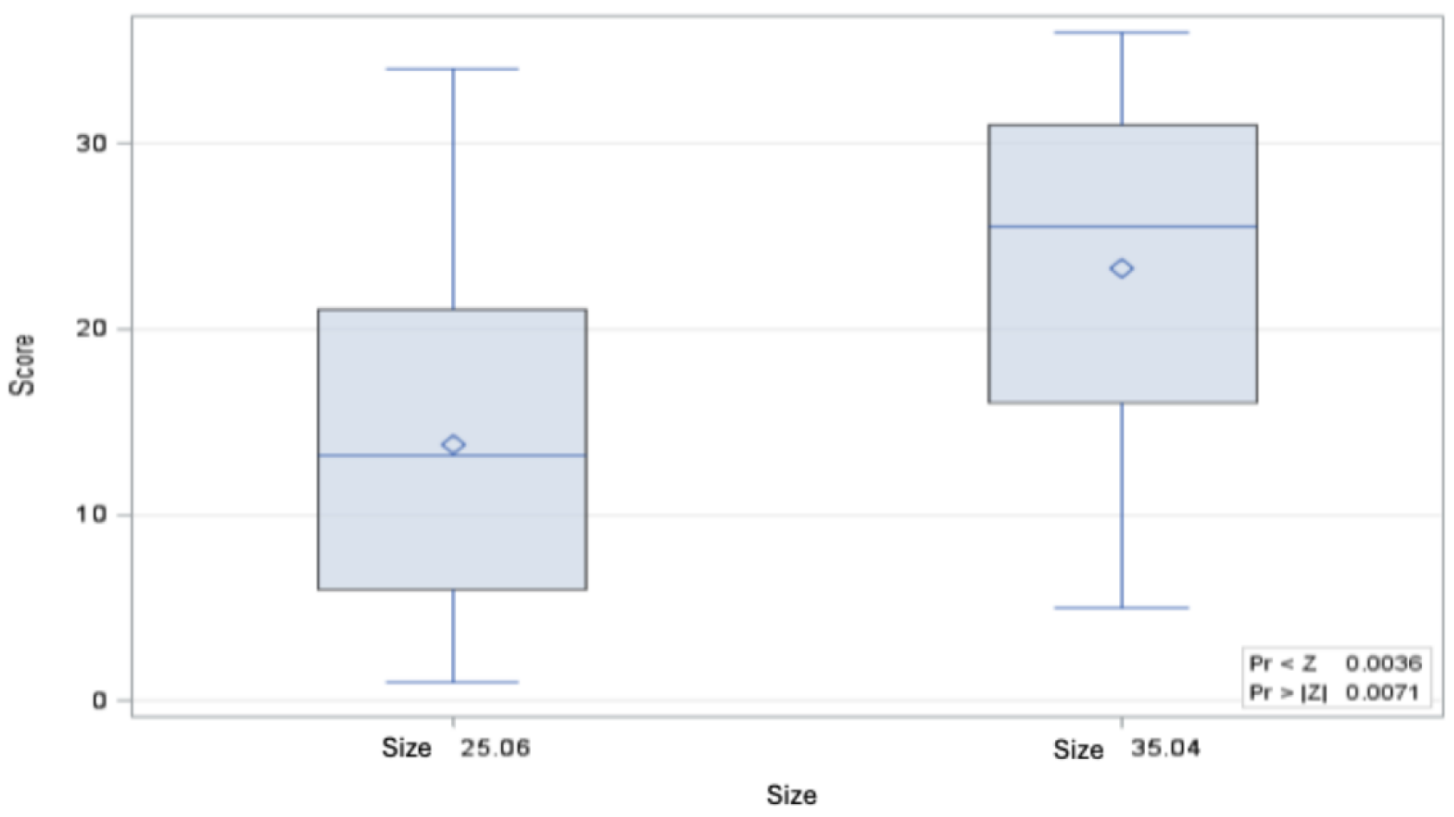
Distribution of Wilcoxon score values for the bending moment of size 35.04 untreated vs. 12 h immersion in 6 M GdnSCN solution (p=0.0490) (minimum, 25^th^ percentile, 50^th^ percentile, 75^th^ percentile, maximum and mean value ◊)

When comparing the bending moments of the root canal instruments of both sizes, the bending moment of size 35.04 is significantly greater than that of size 25.05, both in the control and after 8 immersions in 6 M GdnSCN solution for 15 min each and after a single immersion for 12 h in 6 M GdnSCN solution (Figure 3 (Fig. 3), Figure 4 (Fig. 4), and Figure 5 (Fig. 5)).

When comparing the files with size 35.04 and with size 25.06, only size 25.06 showed a significant difference between the control and immersion for 12 h in 5 M GdnSCN solution (p<0.05).

### Fracture behavior

**Figure 6:**
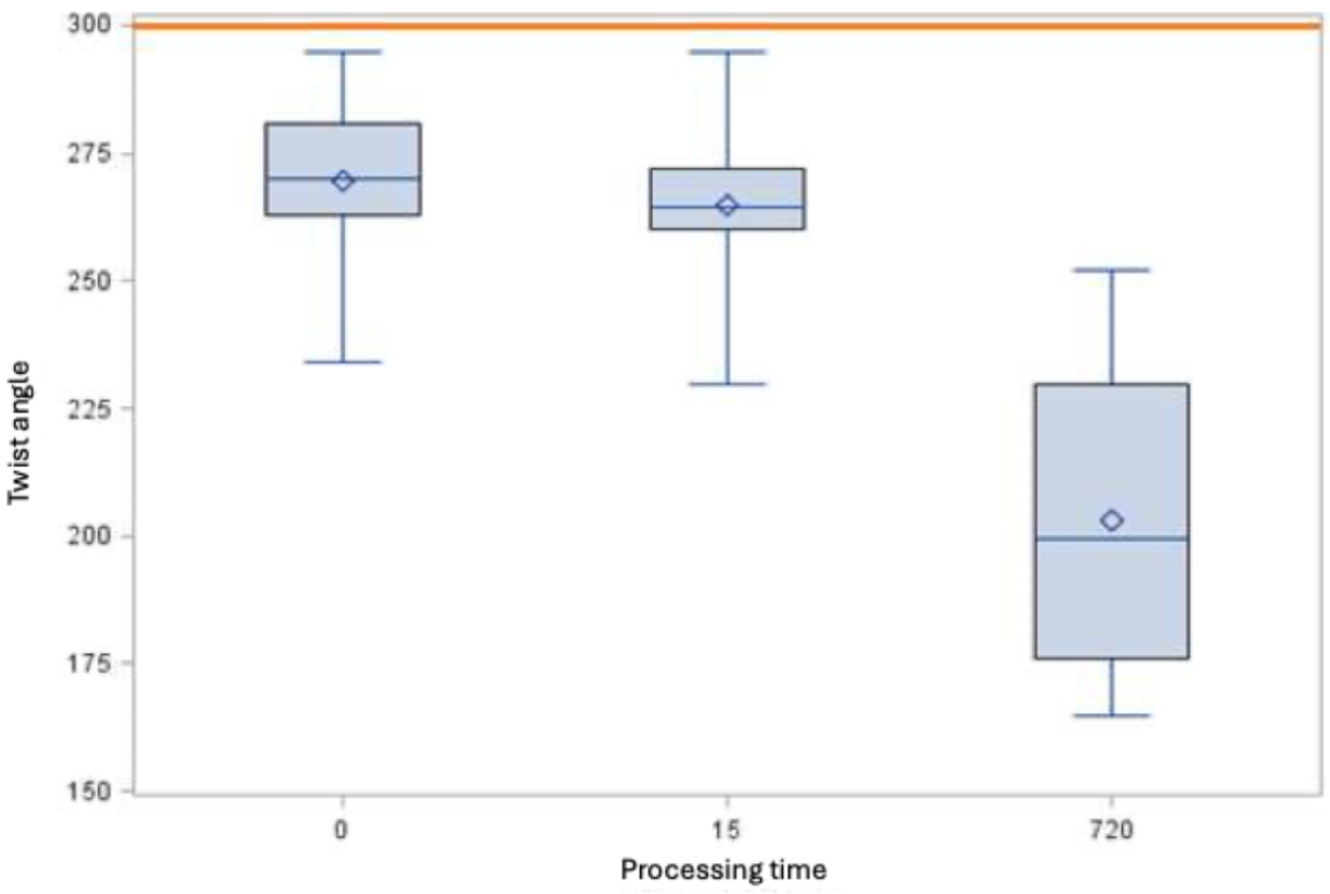
Torsion angle [°] of root canal instruments of size 25.06 (minimum, 25^th^ percentile, 50^th^ percentile, 75^th^ percentile, maximum and mean value ◊, the horizontal red line indicates the derived minimum requirement of 289.8°)

**Figure 7:**
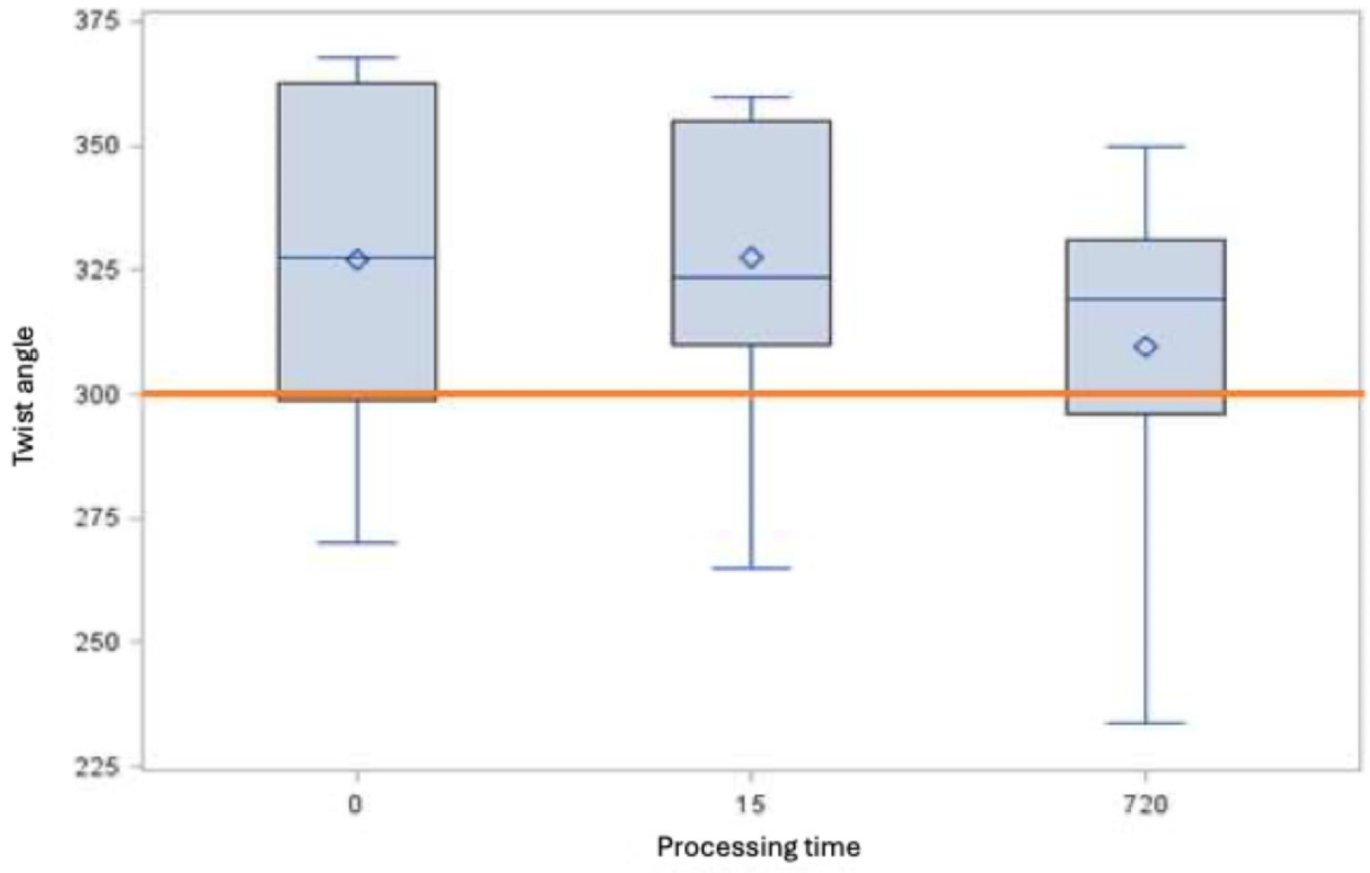
Torsion angle [°] of root canal instruments of size 35.04 (minimum, 25^th^ percentile, 50^th^ percentile, 75^th^ percentile, maximum and mean value ◊, the horizontal red line indicates the derived minimum requirement of 300°)

With regard to fracture behavior, DIN EN ISO [15] does not specify any minimum requirements for rotary nickel-titanium root canal instruments with a taper >2%. If the minimum requirements with regard to the torsion angle are compared with the minimum requirements defined in DIN EN for instruments with a conicity of 2%, the root canal instruments of size 25.06 do not achieve a twisting of 300° either in the control or after immersion in the 5 M GdnSCN solution (Figure 6 (Fig. 6)). In contrast, size 35.04 root canal instruments fulfill the requirements (Figure 7 (Fig. 7)).

### Fracture behavior 

**Figure 8:**
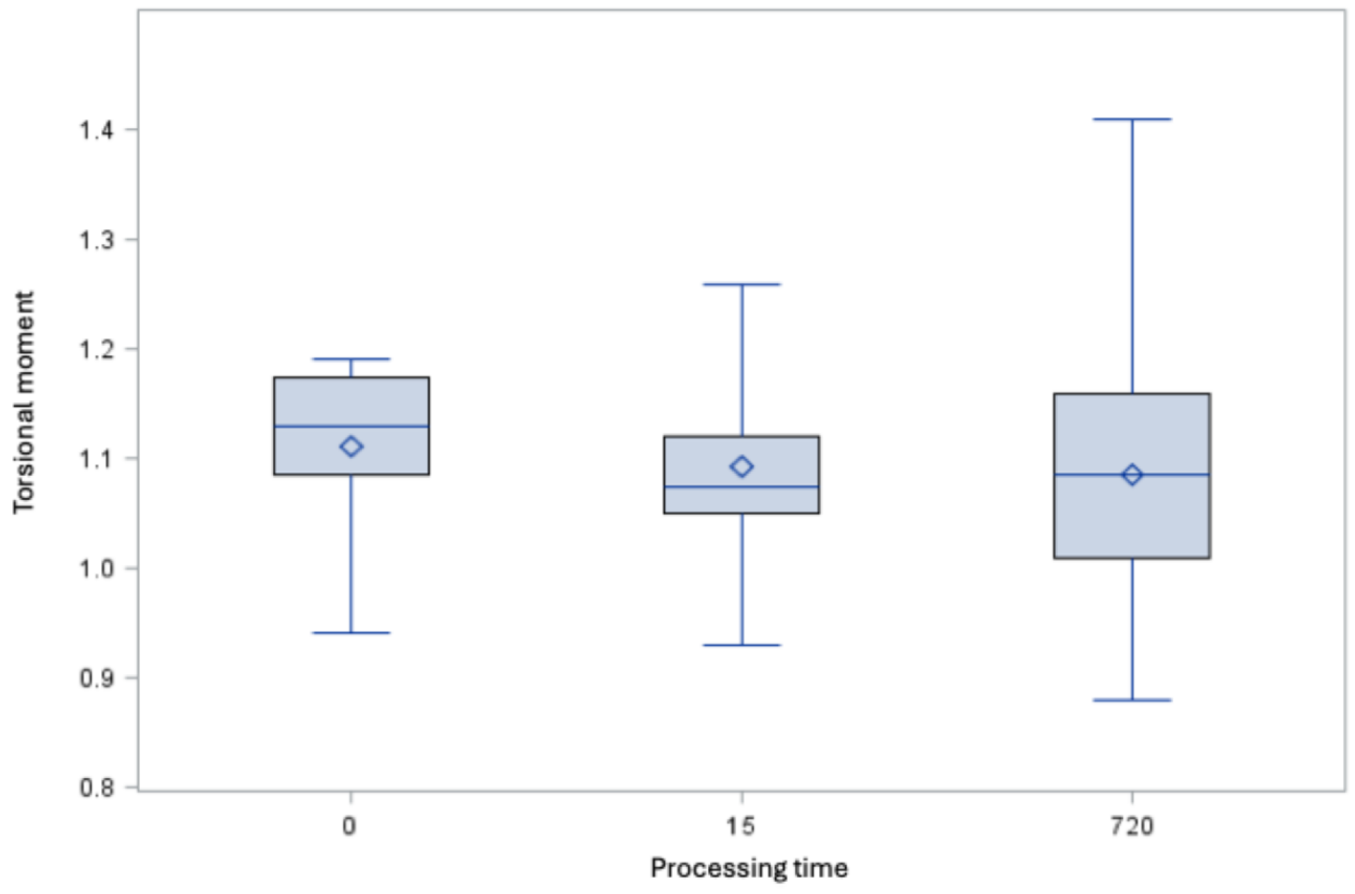
Torsional moment [Ncm] of the root canal instruments of size 25.06 (minimum, 25^th^ percentile, 50^th^ percentile, 75^th^ percentile, maximum and mean value ◊, derived minimum requirement 0.23 Ncm)

**Figure 9:**
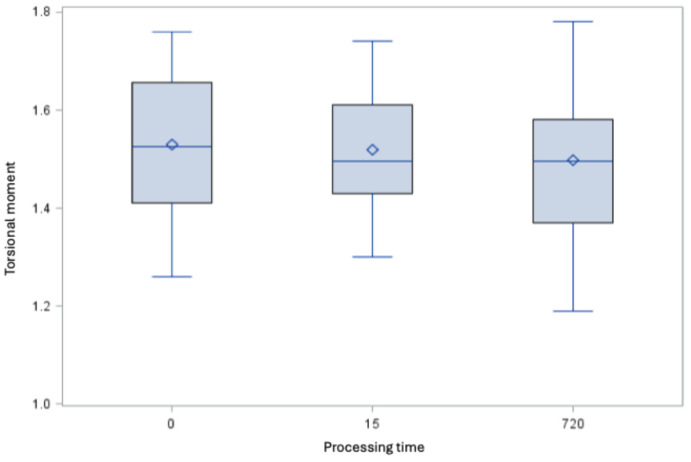
Torsional moment [Ncm] of the root canal instruments of size 35.04 (minimum, 25^th^ percentile, 50^th^ percentile, 75^th^ percentile, maximum and mean value ◊, derived minimum requirement 0.55 Ncm)

The root canal instruments fulfill the requirements with and without treatment with 5 M GdnSCN solution with not significant difference (Figure 8 (Fig. 8) and Figure 9 (Fig. 9)).

## Discussion

### Bending moment 

A lower bending moment means greater flexibility of the files, a desirable quality. Untreated endodontic instruments as well as root canal instruments treated with 5 M GdnSCN solution both fulfilled the requirements for the bending moment without exception and are below the maximum values (Table 1 (Tab. 1)). 

When comparing the bending moments, the size-35.04 files exhibited the greater bending moment (p<0.05, Table 1 (Tab. 1)). They therefore have a lower flexibility and a higher bending stiffness than size-25.06 instruments.

Within instrument sizes 35.04 and 25.06, there was no significant difference in the bending moment between insertion 8 times for 15 min or for 12 h in 6 M GdnSCN solution and untreated instruments, i.e., the flexibility was not influenced by GdnSCN. Only the bending moment of the size-25.05 instruments placed in 5 M GdnSCN solution for 12 h decreased by 6%, associated with an increase in instrument flexibility. However, due to the different clamping possibilities of the instruments over their larger and smaller cross-sections, along with resulting deviations for the values of the bending moment of up to 12% (shown by Soyka [17]), the deviation of 6% is obviously not critical.

Due to the increased flexibility compared to root canal instruments made of stainless-steel alloys, nickel-titanium root canal instruments are able to prepare highly curved root canals largely without undesirable changes in shape [18]. The modulus of elasticity of the nickel-titanium alloy was only 1/5 of the corresponding value for chromium-nickel steel. The higher flexibility leads to less stress on the cutting edges of the instrument in curved root canals and reduces the risk of fracture [19]. The superelasticity of nickel-titanium alloys enables better adaptation to the original course of the canal during root canal preparation and prevents it from being straightened; this means that the canals can be prepared more centrally than is possible with corresponding stainless steel instruments.

Schäfer et al. [19] showed that nickel-titanium instruments with conicities greater than 2% or 4% exhibit less flexibility. This study result could not be confirmed on the basis of the values determined here. The mean values and scatter of the bending moments of the examined instruments of size 35.04 with a taper of 4% were greater (1.005+0.068) than those of the instruments of size 25.06 with 6% taper (0.909+0.053). This result was seen in both the untreated and the GdnSCN-treated instruments.

### Torsional behavior 

DIN EN ISO [15] describes the torsional behavior of root canal instruments in terms of breaking strength and torsion angle. The latter indicates the angle at which an instrument fractures when clamped at its tip and twisted clockwise. The breaking strength indicates the maximum torque achieved during torsion at the moment of fracture. The torsion angles of nickel-titanium instruments are the same order of magnitude as those of comparable stainless-steel instruments. In contrast, the values for fracture resistance are lower than those of analog stainless-steel instruments. Nickel-titanium instruments exhibit fundamentally different fracture behavior than stainless steel reprocessing instruments, which can be explained by the pseudo-elastic behavior of nickel-titanium alloys. Instruments made of stainless-steel exhibit a wide range of plastic deformation under load, which usually appears macroscopically as permanent torsion. In contrast, there is no macroscopically visible deformation (untwisting of the twist) in nickel-titanium instruments. The fracture behavior of nickel-titanium instruments is therefore of great clinical relevance.

The torsion angles of the untreated instruments and those treated with 5 M GdnSCN solution do not differ for size 35.04 (p>0.05). For size 25.06, the torsion angle differs from the control only after 12 h immersion in 5 M GdnSCN solution (p<0.05). Consequently, the manufacturer’s recommendation of an exposure time of 15 min instead of 12 h should be adhered to, in order avoid material damage (Table 2 (Tab. 2)).

The order of magnitude of torsion angles of the nickel-titanium instruments is comparable to that of stainless-steel instruments, while the values for breaking strength are significantly lower than those of analogous stainless steel instruments (Table 3 (Tab. 3)). This can be explained by the pseudo-elastic behavior of nickel-titanium alloys. Stainless steel instruments exhibit a wide range of plastic deformation under load, which macroscopically usually appears as permanent torsion. In contrast, a macroscopically visible deformation (untwisting of the twist) is not recognizable in nickel-titanium instruments.

### Limitations

When testing the bending moment in accordance with DIN EN ISO 3630-1:2008-04 [15], the instrument is clamped between two brass jaws to a depth of 3 mm starting from the tip of the instrument and then bent up to 45°. However, the standard [15] does not describe or specify how the bending moment is to be measured depending on the design of the instrument to be tested. Since the EasyShape^®^ instruments tested have an S-shaped or rhomboid-like cross-sectional shape with different side lengths, there are two ways of clamping the instrument between the jaws, taking the instrument cross-section into account. As a result, the bending behavior can be tested using either the smaller or the larger instrument diameter, which has an influence on the level of the bending moments [17]. The influence of the orientation of the instrument in the testing device (bending over the largest and smallest cross-section) on the bending moment could be confirmed and differs significantly (Kruskal-Wallis test p=0.0014). The difference between the arithmetic mean values is approx. 10%. Since the cross-sectional geometry of the files in conjunction with the type of positioning of the clamping jaws influences the level of the bending moment to be measured, the measured bending moment is less suitable for comparative investigations. However, as the measurements in the study were only carried out over the smaller instrument diameter, the results between control and treatment with GdnSCN are comparable.

In contrast to the bending moment, the various clamping options for the instruments in the testing machine are only of secondary importance for torsional movements of the files [17]. 

Apart from the manufacturer’s recommended maximum of 8 decontaminations with 5 M GdnSCN solution for 5 min each, not only the effect of GdnSCN, but also the mechanical stress with possible material fatigue and the thermal stress caused by steam sterilization can have an influence, although no influence on fracture behaviour was found for the sterilization process [18]. Nevertheless, it is important to clarify what influence the overall reprocessing procedure has on the performance of the instruments. 

## Conclusions

Decontamination with GdnSCN has no effect on the torsional strength or flexural rigidity of nickel-titanium root canal instruments of sizes 25.06 and 35.04 when used properly in accordance with the manufacturer’s instructions (8 immersions for 15 min each). 

However, if the exposure time exceeds 12 h, the bending moment and the torsion angle of instrument size 25.06 are reduced. This may result in the files fracturing sooner than untreated instruments in the event of becoming lodged in the root canal.

## Notes

### Competing interests

The authors declare that they have no competing interests.

### Funding

None. 

### Acknowledgement

We would like to thank Gebrüder Brasseler GmbH & Co, in particular Mr. Michael Krumsiek, for the opportunity to carry out tests in the product development laboratory, the constructive advice and the great willingness to help. We would also like to thank Isabell Gornik for her friendly support in the development laboratory.

### Authors’ ORCIDs

- Kramer A: https://orcid.org/0000-0003-4193-2149
- Schulz-Schaeffer WJ: https://orcid.org/0000-0001-5886-2322

## Figures and Tables

**Table 1:**
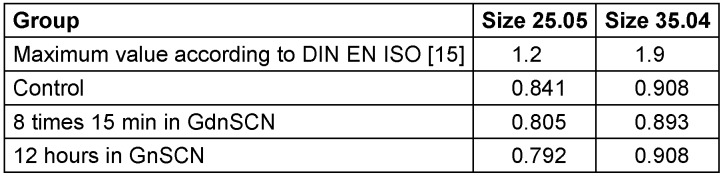
Mean values of the bending moment

**Table 2:**
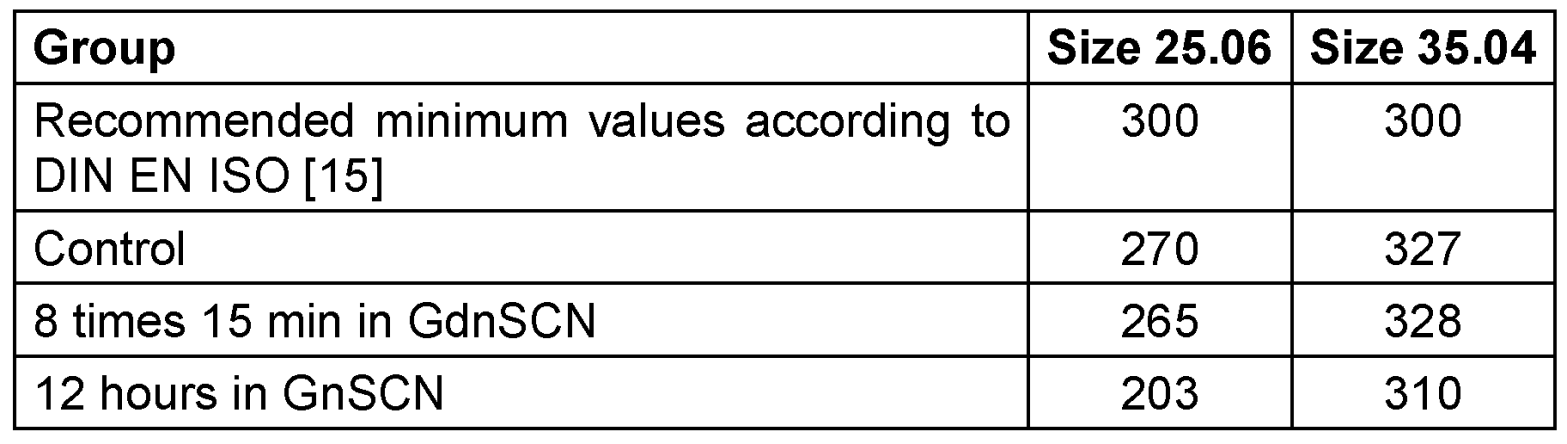
Mean values of the torsion angle [°]

**Table 3:**
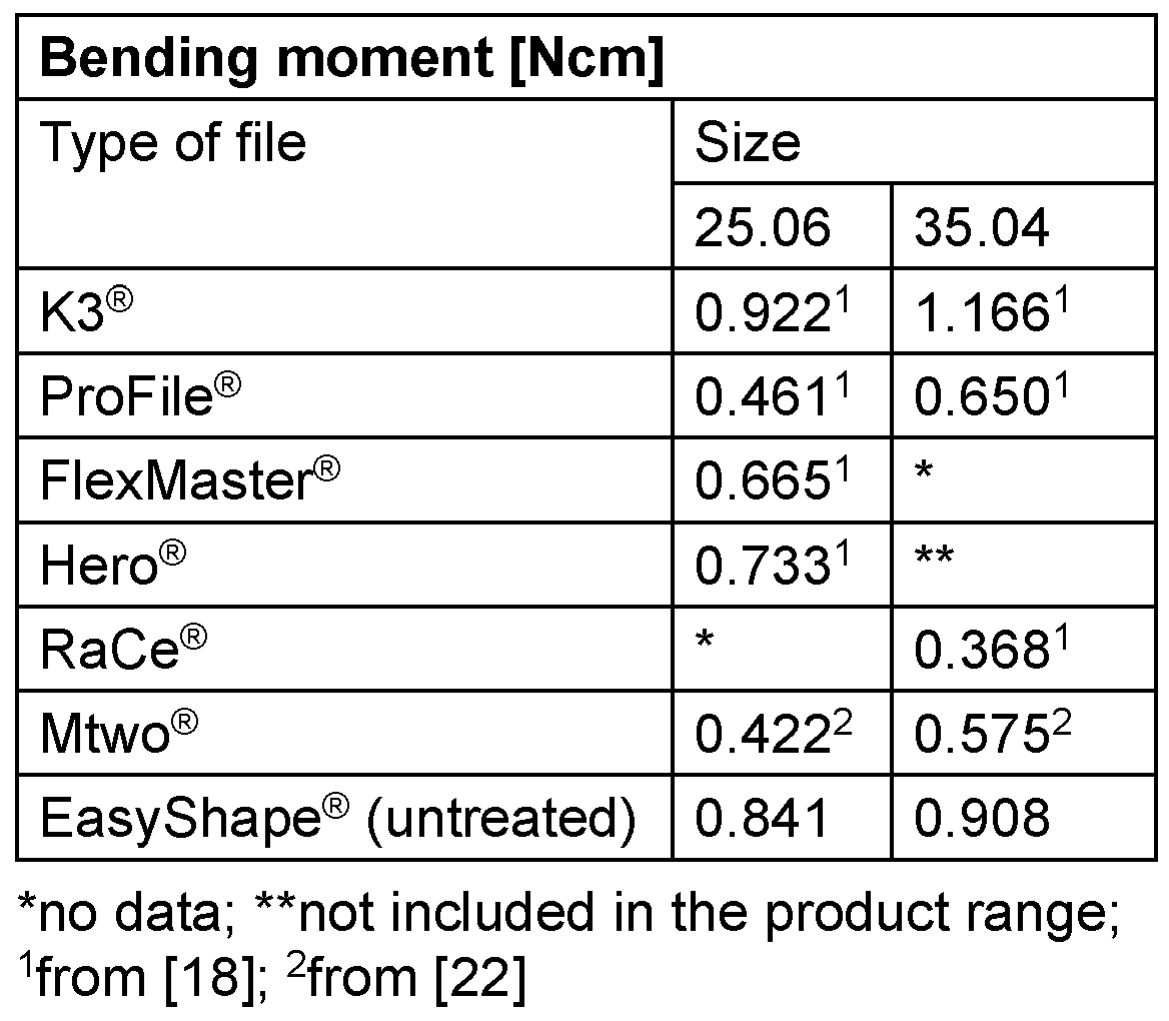
Comparison of the values for the bending moment from the study by Dzepina [21] and Soyka [17] with the values for EasyShape^®^
